# Supplementary material for: The biophysical basis underlying the maintenance of early phase long-term potentiation
Source: PLoS Comput Biol. 2021 Mar 22;17(3):e1008813. doi: 10.1371/journal.pcbi.1008813 (PMC8016278; doi:10.1371/journal.pcbi.1008813)
Supplement: S1 Fig — For the three models of AMPAR-dynamics (first column: basic model; second column: sLTP model; third column: cooperative binding model), the exocytosis event rate under basal condition (kexo0; first row), the maximal amplitude of LTP-induced exocytosis event rate (kexoA=kexo0(1+Aexo); second row), and the decay time of the exocytosis event rate after LTP-induction (τexo; third row) are shown. Black lines depict standard deviations. The red areas indicate the biologically plausible regime of the corresponding indicators based on experimental studies [11, 12, 47]. (PDF) [file pcbi.1008813.s001.pdf]

S1 Figure

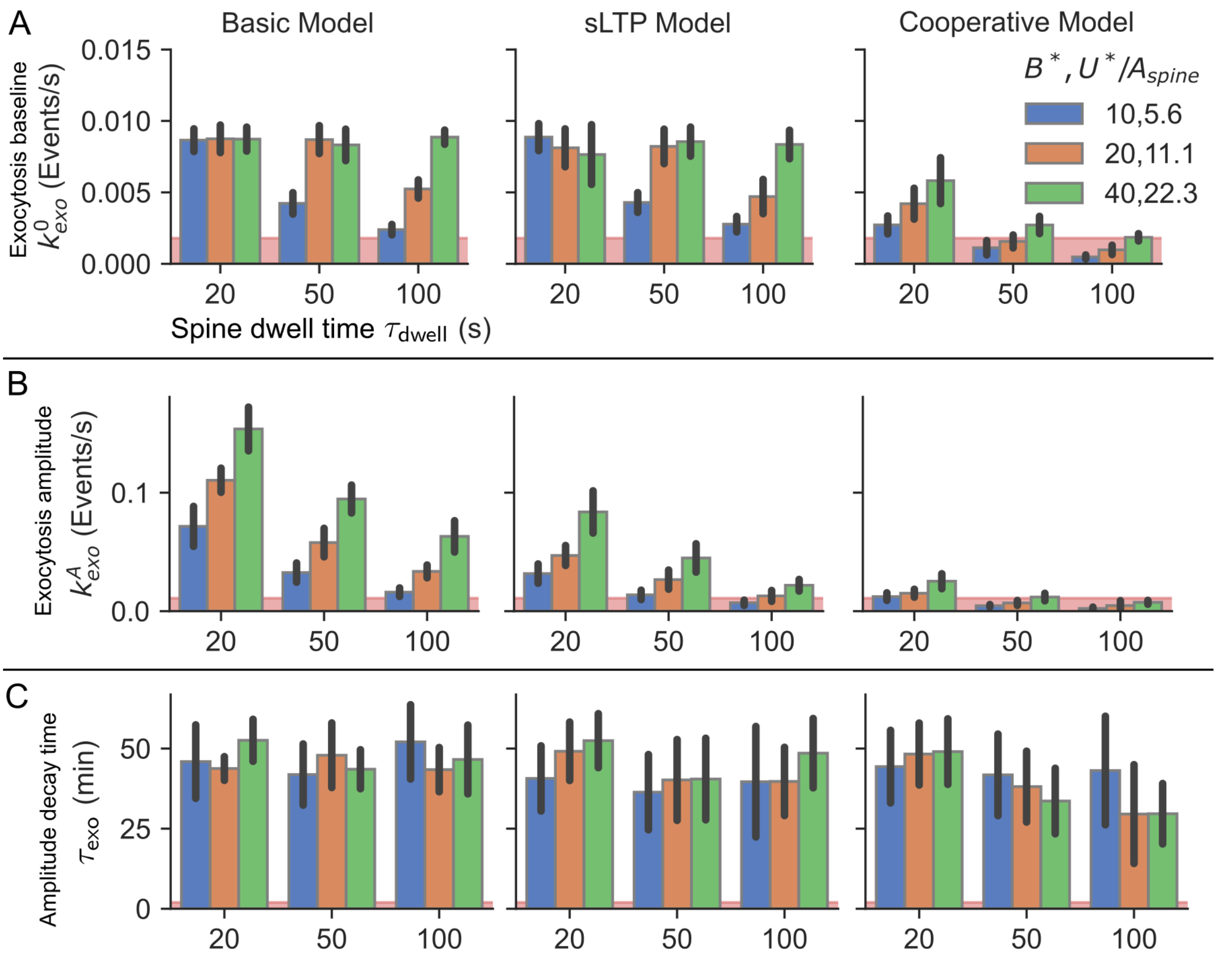

S1 Fig. The parameter values of exocytosis for the best 0.5% parameter sets depend on the number of bound AMPARs under basal condition and the dwell time of mobile receptors at the spine. For the three models of AMPAR-dynamics (first column: basic model; second column: sLTP model; third column: cooperative binding model), the exocytosis event rate under basal condition (k^0_{exo}; first row), the maximal amplitude of LTP-induced exocytosis event rate ($k^A_{exo} = k^0_{exo}(1 + A_{exo})$; second row), and the decay time of the exocytosis event rate after LTP-induction (τ_{exo}; third row) are shown. Black lines depict standard deviations. The red areas indicate the biologically plausible regime of the corresponding indicators based on experimental studies [1, 2, 3].

References

[1] Passafaro M, Piëch V, Sheng M. Subunit-specific temporal and spatial patterns of AMPA receptor exocytosis in hippocampal neurons. Nature Neuroscience. 2001;4(9):917–926. doi:10.1038/nn0901-917.

[2] Yudowski GA, Puthenveedu MA, Leonoudakis D, Panicker S, Thorn KS, Beattie EC, et al. Real-Time Imaging of Discrete Exocytic Events Mediating Surface Delivery of AMPA Receptors. Journal of Neuroscience. 2007;27(41):11112–11121. doi:10.1523/jneurosci.2465-07.2007.

[3] Patterson MA, Szatmari EM, Yasuda R. AMPA receptors are exocytosed in stimulated spines and adjacent dendrites in a Ras-ERK-dependent manner during long-term potentiation. Proceedings of the National Academy of Sciences. 2010;107(36):15951–15956. doi:10.1073/pnas.0913875107.
