# Supplementary material for: The biophysical basis underlying the maintenance of early phase long-term potentiation
Source: PLoS Comput Biol. 2021 Mar 22;17(3):e1008813. doi: 10.1371/journal.pcbi.1008813 (PMC8016278; doi:10.1371/journal.pcbi.1008813)
Supplement: S1 Appendix — (PDF) [file pcbi.1008813.s002.pdf]

S1 Appendix

Exocytic factor

As mentioned in the main text, there are other possible explanations for the dependence of E-LTP on exocytosis. One hypothesis states that due to AMPAR type specific binding affinities, exocytosis of a different AMPAR type is necessary for E-LTP expression [1]. In the following, we introduce another version of our model extended by such mechanisms and show that the resulting dynamics also match experimental results. We introduce two AMPAR types each having its own trafficking properties. These different properties may originate from a different subunit composition or phosphorylation state [1, 2].

$$\frac{dU^I}{dt} = k_{exo}\, S^I_{exo} + k^I_{in} + k^I_{BU}\, B^I - \left(k_{endo} + k_{out} + k^I_{UB}(P - (B^I + B^{II}))\right) \frac{U^I}{A_{spine}}. \quad (1)$$

$$\frac{dB^I}{dt} = k^I_{UB}(P - (B^I + B^{II}))\, \frac{U^I}{A_{spine}} - k^I_{BU} B^I. \quad (2)$$

$$\frac{dU^{II}}{dt} = k_{exo}\, S^{II}_{exo} + k^{II}_{in} + k^{II}_{BU}\, B^{II} - \left(k_{endo} + k_{out} + k^{II}_{UB}(P - (B + B^{II}))\right) \frac{U^{II}}{A_{spine}}. \quad (3)$$

$$\frac{dB^{II}}{dt} = k^{II}_{UB}(P - (B^I + B^{II}))\, \frac{U^{II}}{A_{spine}} - k^{II}_{BU} B^{II}. \quad (4)$$

We assume four things:

1. Exocytosis event rates, endocytosis rates and diffusion of the two receptor types are the same;
2. receptor type II enters the spine only via exocytosis, not diffusion, i.e. we set $k^{II}_{in} = 0$;
3. receptor type II has an increased binding affinity, i.e. $k^{II}_{UB} > k^I_{UB}$ and $k^{II}_{BU} < k^I_{BU}$;
4. the second receptor pool is only available after LTP induction by a transition of the intracellular pool from receptor type I to type II.

The latter assumption is modeled by introducing the second receptor type to the intracellular pool (S_{exo}) in the following way:

$$\frac{dS^I_{exo}}{dt} = k^{RE}_{in} - k^{RE}_{out}\, \frac{S^I_{exo}}{V_{spine}(t)} - k_{I-II}\, S^I_{exo} + k_{II-I}\, S^{II}_{exo}, \quad (5)$$

$$\frac{dS^{II}_{exo}}{dt} = -k^{RE}_{out}\, \frac{S^{II}_{exo}}{V_{spine}(t)} + k_{I-II}\, S^I_{exo} - k_{II-I}\, S^{II}_{exo}, \quad (6)$$

where k_{I-II} and k_{II-I} are the transition rates from type I to type II and vice versa. Such a transition could reflect a long-lasting change e.g. in the receptors phosphorylation

state due to increased CaMKII activity or a change in AMPAR subunit composition [3, 4]. However, the role of AMPAR phosphorylation in regulating receptor binding at the PSD is still unclear [5]. Parameter values are shown in Table 1 and Table 2 in S1 Appendix. All other parameters and the binding/unbinding rates for type I are the same as in the main text. Also, the binding rate increase during LTP induction for receptor type II is taken to be the same as for type I. Cooperative receptor binding and an sLTP mediated increase in exocytosis event size S_{exo} are not accounted for but spine volume increase is. In this model, E-LTP is maintained under normal conditions whereas rapidly decaying in the absence of exocytosis (see Fig 1 in S1 Appendix).

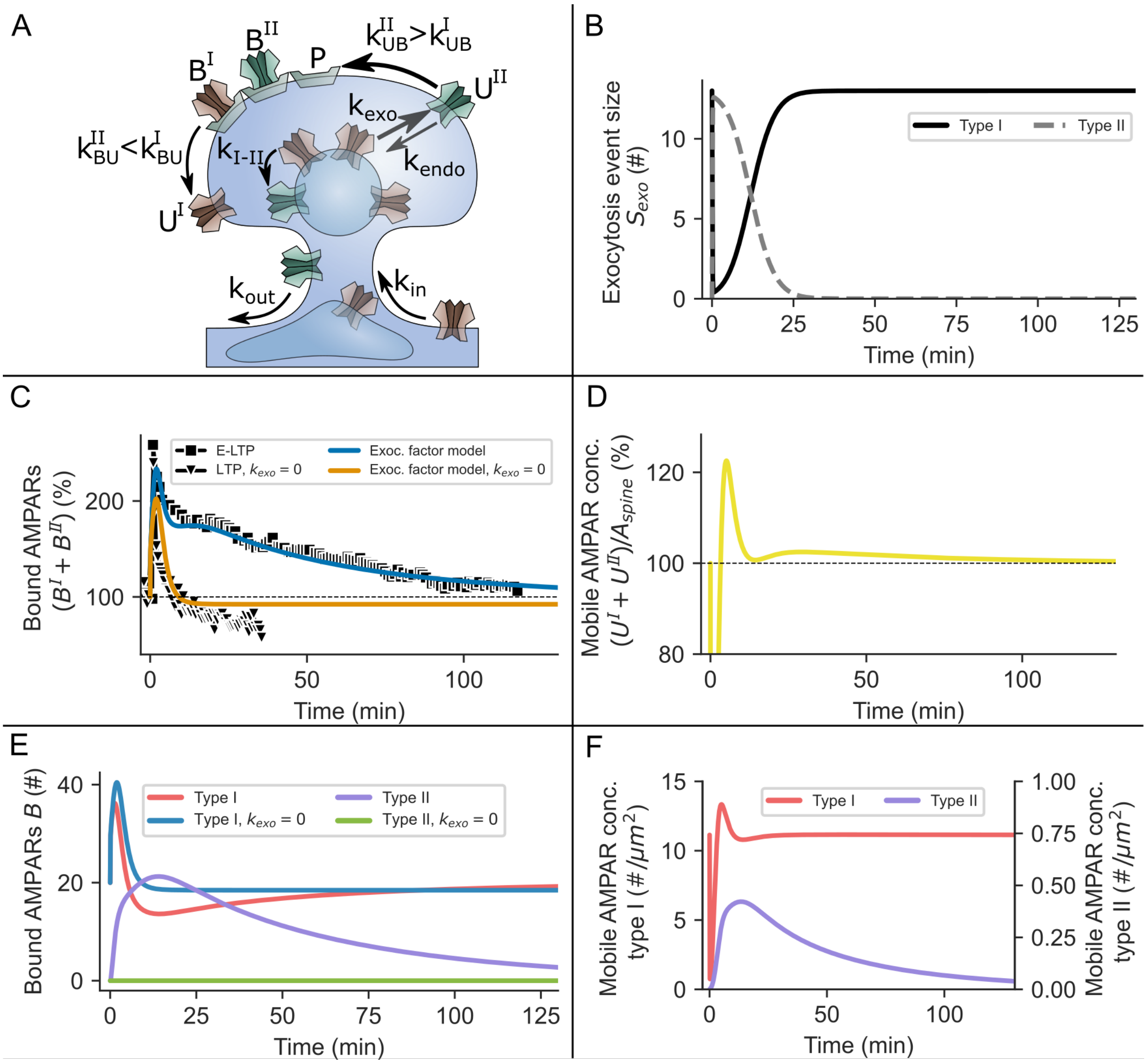

Fig 1. E-LTP is maintained by exocytosis of a second AMPAR type having a higher binding affinity. (A) A second AMPAR type (green) with different binding and unbinding rates k_{UB}^{II}, k_{BU}^{II} is introduced. The availability of the second type is regulated intracellular. Receptors available for exocytosis in the intracellular pool (S_{exo}) can transition between these two types at rates k_{I-II}, k_{II-I}. **(B)** Before LTP induction, the intracellular pool for receptors to become exocytosed consists only of type I AMPARs.

At the time point of LTP induction (t=0), k_{I-II} is transiently increased such that type I AMPARs (black line) are exchanged by type II AMPARs (grey line). As the transition rate k_{I-II} decays, the intracellular type II pool slowly depletes, whereas the type I pool is gradually replenished. **(C)** The transition to the exocytosis of type II AMPARs, having an increased binding affinity, mediates the expression of E-LTP (blue line). In the absence of exocytosis, type II AMPARs are absent at the spine membrane, resulting in a rapid decay of LTP (experimental data taken from [6, 7]). **(D)** The concentration of mobile receptors ($U = U^{II} + U^{I}$ rapidly increases due to the rise in the exocytosis event rate. **(E)** The initial increase in bound receptors is mediated by the rapid trapping of type I AMPARs already present at the spine surface (red and blue (blocked exocytosis) lines). Subsequent exocytosis of type II AMPARs results in a small drop of bound type I AMPARs and the accumulation of type II at the PSD (purple line). In the absence of exocytosis, no type II AMPARs are bound (green line).**(F)** The concentration of mobile type I AMPARs initially drops due to the rapid increase in spine volume and binding rate but quickly recovers (red line). As type II AMPARs are exocytosed, their concentration increases but decays as the intracellular transition from type I to type II goes to zero **(B)**.

Table 1. Parameters of AMPAR trafficking with two receptor types.

Parameter	Meaning	Value
k_{in}^{II}	Rate at which AMPARs enter the spine via lateral diffusion	0.0 $\#/s$
k_{BU}^{II}	Unbinding rate of receptors from PSD slots	0.025 s^{-1}
$k_{UB}^{II,0}$	Binding rate of AMPARs at the PSD	0.036 $\mu m^2/(\#s)$
k_{I-II}	Transition rate from type I to type II	0.0 s^{-1}
k_{II-I}	Transition rate from type II to type I	0.0 s^{-1}

Table 2. LTP-induced parameter changes (two type model).

Parameter	Meaning	Value
τ_{I-II}^{1}	Time constant of I-II transition rate increase during LTP induction	5 s
τ_{I-II}^{2}	Decay time constant of I-II transition rate after LTP induction	180 s
A_{I-II}	Amount by which the transition rate increases during LTP-induction	0.25 s^{-1}

References

[1] Diering GH, Huganir RL. The AMPA Receptor Code of Synaptic Plasticity. Neuron. 2018;100(2):314–329. doi:10.1016/j.neuron.2018.10.018.

[2] Hiester BG, Becker MI, Bowen AB, Schwartz SL, Kennedy MJ. Mechanisms and Role of Dendritic Membrane Trafficking for Long-Term Potentiation. Frontiers in Cellular Neuroscience. 2018;12. doi:10.3389/fncel.2018.00391.

[3] Huganir RL, Nicoll RA. AMPARs and Synaptic Plasticity: The Last 25 Years. Neuron. 2013;80(3):704–717. doi:10.1016/j.neuron.2013.10.025.

[4] Lee SJR, Escobedo-Lozoya Y, Szatmari EM, Yasuda R. Activation of CaMKII in single dendritic spines during long-term potentiation. Nature. 2009;458(7236):299–304. doi:10.1038/nature07842.

[5] Choquet D. Linking Nanoscale Dynamics of AMPA Receptor Organization to Plasticity of Excitatory Synapses and Learning. The Journal of Neuroscience. 2018;38(44):9318–9329. doi:10.1523/jneurosci.2119-18.2018.

[6] Penn AC, Zhang CL, Georges F, Royer L, Breillat C, Hosy E, et al. Hippocampal LTP and contextual learning require surface diffusion of AMPA receptors. Nature. 2017;549(7672):384–388. doi:10.1038/nature23658.

[7] Barco A, Alarcon JM, Kandel ER. Expression of Constitutively Active CREB Protein Facilitates the Late Phase of Long-Term Potentiation by Enhancing Synaptic Capture. Cell. 2002;108(5):689–703. doi:10.1016/s0092-8674(02)00657-8.
