# Supplementary material for: The biophysical basis underlying the maintenance of early phase long-term potentiation
Source: PLoS Comput Biol. 2021 Mar 22;17(3):e1008813. doi: 10.1371/journal.pcbi.1008813 (PMC8016278; doi:10.1371/journal.pcbi.1008813)
Supplement: Attachment — Submitted filename: Response_to_reviewers.pdf [file pcbi.1008813.s003.pdf]

List of changes:
The biophysical basis for early phase long-term potentiation

Moritz Becker Christian Tetzlaff

January 30, 2021

We would like to thank both referees for their questions and detailed comments. Due to these comments, we have rewritten parts of the manuscript to make our results more clear. We have revised figures 6A, 7H and 3C and extended the discussion, which now also includes sections on signaling pathway models, the location of exocytosis and the role of exocytic factors. Also, we included analysis of an alternative model that is based on AMPAR type specific binding affinities in the supplement.

We hope that the manuscript is now in an adequate state.

With kind regards,
Moritz Becker and Christian Tetzlaff

Referee 1

Major points:

1. `Referee: I think that there is a mistake in the number of slots P of table 1 (70) because after the simulation of LTP the number of bound AMPARs is more than 200 (Fig. 2). If I understood well, the number of slots is not changed during the simulation of E-LTP, and there are always slots in excess with respect to the number of bound receptors.`

   This is actually not a mistake, as the number of bound receptors in Fig 2 is given in "% of the baseline" for better comparison to experimental data (also often given in percent of the baseline level). To avoid confusion, we have now added a sentence to make clear that in the figures, AMPAR numbers are given in % most of the time.

   > (IN PAPER, page 9:) [...] To examine whether a brief increase in exocytosis event and binding rate can account for the maintenance of E-LTP for several hours as well as the fast decay in the absence of exocytosis, we first study a basic model of AMPAR trafficking. Note that we compare bound AMPARs to EPSP data, which is given in percentage of the baseline value. Also, many experimental methods do not yield quantitative data on receptor numbers, e.g. fluorescence microscopy. Therefore, in this study, receptor numbers are shown in percentage of the baseline level, if not stated otherwise. [...]

2. `Referee: Again regarding P values, they refer to the total number of slots of the synapse or the number of slots of a nanodomain? Several studies have revealed the presence of at least 2-3 nanodomains 70 nm-wide in a subset of synapses, each of these nanoclusters containing 20 receptors (i.e. Nair et al 2013) so a P value of 25 would only represent one nanodomain but not the entire PSD.`

   The model only accounts for a single nanodomain of scaffolding proteins on which, however, several AMPAR nanodomains/cluster can form. Thus, P refers to the total number of slots in the synapse. As you stated, experiments show that the number of AMPAR nanodomains can range from 0-6 with on average 2.5 domains per spine (Nair et al 2013). However, we argue that the number of slots P is related to the scaffolding proteins, such as PSD95. The majority of spines seem to form one PSD95 domain per synapse, whereas larger spines can have up to 5 (Nair 2013, Hruska 2018). Therefore, one domain should be a good approximation for small spines. The number of receptors being located at the PSD (20) has been estimated based on different studies, but only represents an average value across different synapses with some synapses having much higher receptor numbers. We have now added a sentence in the revised manuscript to clarify that we only considering a single domain formed by scaffolding proteins and that P therefore represents the total number of binding sites.

   > (IN PAPER, page 12:)
   >
   > [...] In the mean-field model, cooperativity results in effective binding and unbinding rates k_{UB}^{coop}, k_{BU}^{coop} that depend on the number of already bound receptors B as well as the number of binding sites P (Fig 3B). Without loss of

generality, we assume that the synapse contains a single domain of scaffolding proteins; therefore, P represents the total number of binding sites at the synapse. [...]

3. `Referee: Regarding the rates of AMPARs entering (kin) or exiting (kout) the spine, they are not changed even after AMPAR exocytosis, is this right? But could we expect that the increase in the number of membrane receptors in the spine will shift the equilibrium towards a higher kout?`

   Actually, the effective rate at which receptors exit the spine does change. k_{out} only describes the rate at which receptors exit the spine relative to the receptor concentration. Given equation 1, the whole term $k_{out} \cdot U/A_{spine}$ describes the number of receptors that exit the spine per time step. Thus, as exocytosis yields an increase in U, the total efflux also increases. We have now clarified this point.

   (IN PAPER, page 10) [...] The time-dependence of k_{exo} and k_{UB} is described by an exponential rise (τ^1_{exo}, τ^1_{UB}) and decay (τ^2_{exo}, τ^2_{UB}) with maximum amplitudes of A_{exo} and A_{UB} (see Table 2 and Equations (6)-(8)). Whereas the rates k_{out} and k_{endo} are kept constant, the total efflux via the corresponding trafficking pathways does vary in response to receptor concentration changes (U/A_{spine}) (see Equation (1)). [...]

4. `Referee: In the cooperative model, how the maximum possible number of neighbors was calculated? Is it four as the scheme in Fig. 3A? Does this value come from experimental data?`

   The maximum number of possible nearest neighbors is determined by the spatial grid being assumed. For simplicity, we used a square grid resulting in a maximum number of eight possible neighbors. We have now clarified this point in the revised manuscript.

   (IN PAPER, page 12:) [...] Our model of cooperative AMPAR-dynamics, based on [1], enables us to investigate the influence of cooperative binding effects on the maintenance of E-LTP. In this model, the probability of receptors to bind to or unbind from a slot depends on the number of nearest neighbours on a grid that represents the PSD (Fig 3A). We used a square grid, such that the number of nearest neighbours is at maximum 8. [...]
   (IN PAPER, page 35:) [...] In our adaptation of this model the scaffolding proteins or slots are spatially organized on a square grid (Fig 3A) where each mobile receptor U binds to a free slot with a certain rate, dependent on the number of occupied nearest-neighbor slots (Equation (14)). Note that one could use different types of grids, e.g. a hexagonal grid, to account for a different maximum number of nearest neighbours. [...]

5. `Referee: Why the units of the binding rate are` $\mu m^2/s$? `An explanation of the meaning of this value would help the reader to compare it with values obtained or employed in other studies.`

   Equations 1 and 2 describe the change in receptor numbers while some processes, such as receptor binding, endocytosis and the spine efflux via diffusion depend on the receptor concentration. Thereby, some of the rate constants have units of $\mu m^2/s$.

This is a standard method in physics and mathematical modeling and follows from the requirement for equal units on the left and right hand site of an equation. One may compare this to the definition of the diffusion coefficient in Fick's law of diffusion, where the particle flux is given by the concentration gradient times the diffusion coefficient, which also has units of m^2/s. We have now added a short paragraph in the model introduction where we explain the origin of the different parameter units.

> (IN PAPER, page 7:) [...] Equations (1) and (2) describe the AMPAR-dynamics on the spine's membrane under basal condition. We derived the corresponding set of parameter values of an average spine by estimating the values from several experimental studies that are mainly conducted in hippocampal cultures and slices (see Table 1) and are described in more detail in the methods section. Note that the receptor flux, for example for diffusion out of the spine or for receptor binding, depends on the receptor concentration U/A_{spine}. Thereby, some of the rate constants have units of $\mu m^2/s$. This follows from the requirement for equal units on the left and right hand site of equations (1) and (2). [...]

6. `Referee: Do the authors think that there should be a minimum size for the synapse to undergo an efficient LTP? Indeed, a sigmoid relationship between bound AMPARs/mobile AMPARs is clearly observed only for large synapses. Could this mean that synapses with nanodomains are less efficient to keep receptors inside?`

   We cannot make a clear statement about whether a minimum size is required (of course their needs to exist a sufficient number of free binding sites). However, we do find that synapses with large PSDs are more efficient in keeping receptors than those with small PSDs (Fig 5). This probably originates from the fact that, according to our model, large PSDs are less occupied during LTP induction compared to small PSDs. The higher number of free binding sites in the case of a large PSD thereby promotes rebinding of receptors. Also, whereas the sigmoid relationship between bound AMPARs and mobile AMPARs is also found for small synapses/PSDs, it is less pronounced. Therefore, for the latter, changes in the mobile receptor concentration have a smaller impact on the number of bound receptors as compared to large synapses. However, the latter was not visible in Fig 6A of the original manuscript. We, therefore, changed figure 6A such that it shows the PSD occupancy in percent instead of the number of bound receptors. Having relative instead of absolute quantities now allows for a better comparison between the curves representing different PSD sizes. Also, we now mention the above observations more clearly in the results section on the dependence of E-LTP on spine and PSD size.

   > (IN PAPER, page 18:) [...] Similarly, we find that the system takes longer to return to equilibrium after LTP-induction as we increase the PSD size P while keeping V_{spine} constant and independent of P (Fig 3B). This effect is also present in the non-cooperative model but much less pronounced (not shown). This shows that spines with large PSDs, having more binding sites, are much more efficient in keeping newly inserted receptors if binding is cooperative (Fig 5B,C). We find three indications for why this is the case. First, during LTP induction, large PSDs are less occupied than small PSDs, which promotes rebinding ($max(B)/P \approx 68\%$ for P=40, $\approx 62\%$ for P=100 and $\approx 50\%$ for P=200,

Fig 5B). Second, larger systems take longer to equilibrate in general. Third, even though a sigmoidal dependence between bound and mobile AMPARs is present for different PSD sizes, it is more pronounced for large PSDs (Fig 6A). Thereby, for the latter, a change in the mobile receptor concentration has a stronger impact on the number of bound receptors as compared to small PSDs.

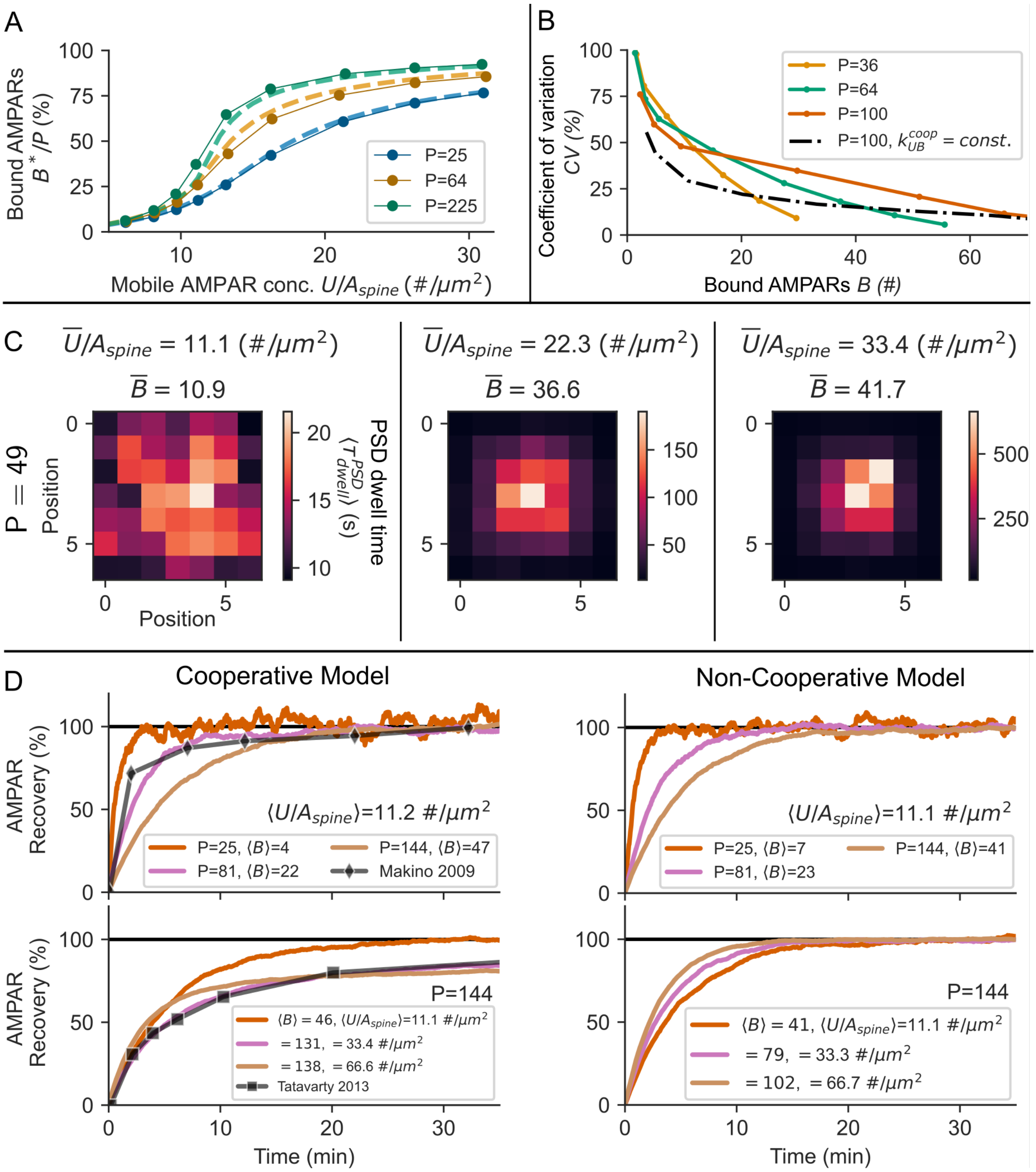

Figure 6. [...] **(A)** Characteristic for cooperative processes, the PSD occupancy in the steady state B^*/P shows a sigmoidal dependence on the concentration of mobile receptors U/A_{spine}, which is maintained for different PSD sizes, but more pronounced for large PSDs. Dots: long term average of stochastic simulations. The standard error of the mean is on the order of the dot size (10 trials). Example traces of B for different mobile receptor concentrations and PSD sizes are found in Fig 7D. Dashed lines: mean-field model. [...]

7. `Referee: The point 3 of the model predictions (lines 316-317) predicts that "receptors are not uniformly distributed in nanodomains... but cluster...". Could the authors define what is a nanodomain and a cluster for them?`

   We agree with the referee that this description is unclear. With nanodomain, we referred to the nanodomain formed by scaffolding proteins. By AMPAR cluster, we referred to the receptors that, due to cooperative effects, do cluster together on a nanodomain of scaffolding proteins. We have now rewritten this part to clarify what we mean.

   > (IN PAPER, pages 21:) [...]
   > (3) Receptors are not uniformly distributed on nanodomains formed by scaffolding proteins but do form smaller cluster on these. Such receptor clustering has been found in experiments [2]. We argue that receptor clustering could be the result of cooperative receptor binding.
   > [...]

Minor points:

1. `Referee: The last sentence in the author summary: "Characterizing the principles... the role of synaptic dynamics in neurodegenerative diseases" is rather out of context, as no other reference exists in the manuscript about these pathologies.`

   Thank you. We agree and have deleted this sentence from the summary.

2. `Referee: Synapses are simulated as occurring in "spines", what is actually the case of excitatory synapses in principal cells. However, the structure "spine" is not described in the introduction. Fig. 2 shows the structure without any explanation. The role of cytoskeleton in shaping the spine should also be mentioned.`

   Thank you for pointing this out. We have now added a short description of the term spine and also added a sentence on the role of the cytoskeleton dynamics. Also, we agree that the description of the spine depiction in Fig 2 is too short and therefore we have adapted the figure caption.

   > (IN PAPER, page 3:) [...] Synaptic plasticity regulates synaptic transmission strength and is associated with cognitive processes such as learning and memory. For excitatory synapses, the transmission strength is mainly determined by the number of α-amino-3-hydroxy-5-methyl-4-isoxazolepropionic acid receptors (AMPARs) accumulating at the postsynaptic density (PSD), which is a dense, membrane-less organelle located within dendritic spines [3, 4, 5, 6]. Dendritic spines are membraneous protrusions that serve as compartments for proteins and organelles involved in synaptic transmission. [...]

   > (IN PAPER, page 5:) [...] However, additional biological and biophysical mechanisms influence the AMPAR dynamics at the dendritic spine. The spine itself is not static but shaped by the dynamics of the spine's cytoskeleton. The induction of LTP also yields structural modifications of the spine (sLTP), which is expressed by an increase in spine volume due to actin polymerization, and

the translocation of AMPAR-containing recycling endosomes (REs) into the spine [7, 8, 9, 10, 11, 12, 13, 14, 15] (Fig 1C). [...]

(IN PAPER, page 9 (Fig 2 caption):) **(A, left)** *Basic model*: AMPA receptors are trafficked across the spine via several pathways. AMPARs are transported from recycling endosomes to the membrane via exocytosis. During this process that occurs at a rate k_{exo}, a number of receptors S_{exo} enters the spine membrane. Similar, AMPARs are inserted into the spine via endocytosis at a rate k_{endo}. In addition, mobile receptors U can laterally diffuse into and out of the spine at rates k_{in} and k_{out}. However, AMPARs only become functional if they are bound to PSD binding sites P. Binding and unbinding occurs at rates k_{UB} and k_{BU}. **(right)** During LTP-induction, exocytosis (k_{exo}) and binding rate (k_{UB}) are briefly increased. The LTP-induced dynamics of the basic model (blue line) is compared to experimental data of E-LTP (black squares; data taken from [16]) and also to LTP with the blockage of exocytosis (orange line compared to black triangles; data taken from [17]). **(B, left)** *sLTP model*: We assume that the number of AMPARs that enter the membrane during an exocytosis event S_{exo} depends on the spine volume and RE trafficking, both of which change during LTP [7, 11]. Therefore, sLTP influences the LTP-induced AMPAR dynamics in addition to k_{exo} and k_{UB}. **(right)** Similar to (A), B increases during LTP induction but only slowly decays back to the baseline if exocytosis occurs. **(C, left)** *Cooperative model*: We hypothesize that binding of receptors is cooperative (red arrows depict receptor-receptor interactions). **(right)** Cooperative receptor binding slows down the decay of LTP induced increase in bound AMPARs and increases the sensitivity to exocytosis. **(D, left and right)** *sLTP+Cooperative Model*: sLTP and cooperative receptor binding in concert with increased exocytosis and binding rate sustain E-LTP . **(A-D)** LTP-induction is triggered at time point $0s$.

3. `Referee: The representations of dwell time in Fig. 6C are difficult to interpret. Which are the units of "Position"?`

   Thank you for pointing this out. We have now added some additional information that hopefully makes this plot easier to understand.

   (IN PAPER, page 20 (Fig 6 caption):) [...] **(B)** In the case of cooperative receptor binding, the CV steeply changes with the size and occupancy of the PSD (colored lines) in contrast to the case in which binding rates are constant (black dotted line).**(C)** Shown is the grid of binding sites for different mobile receptor concentrations. Each pixel depicts a single binding site. The pixel color represents the average dwell time of a receptor in the bound state at that specific binding site (1h time course). As we do not explicitly account for diffusion on the grid, positions are kept unitless.[...]

4. `Referee: A typo: in line 318, replace "too" by "to".`

   Thank you. We have corrected the error.

5. `Referee: Line 390: "GluR1" subunit is named "GluA1" in the current classification of AMPA receptors.`

Thank you for pointing this out. We have now changed GluR to GluA throughout the manuscript.

Referee 2

Major points

1. `Referee: The MS by Becker and Tetzlaff presents a model of synapse function during synaptic plasticity centered on the role of various aspects of the trafficking and stabilization of the main category of glutamate receptors involved in synaptic transmission - i.e. AMPA receptors (AMPAR). Based on a solid and thorough analysis of the experimental data available on the topic, the authors model the contribution of AMPAR exocytosis and endocytosis, synapse growth, AMPAR diffusion and trapping on scaffold protein binding sites, including its potential cooperativity. The authors aim at understanding the potential contribution of these various phenomenon to the "early" phase of activity dependent synaptic potentiation (eLTP) that is observed experimentally and is independent of receptor synthesis. In their model, the authors vary the rates of these various AMPAR trafficking routes and binding affinities, comparing simple and cooperative binding. They compare their simulations The main conclusion of their study is that no single pathway can explain the experimentally observed eLTP but that a combination of increased AMPAR exocytosis and cooperative trapping can reproduce the experimental observations. Altogether this is a solid and thorough study of the potential contribution of various AMPAR trafficking pathways to synaptic potentiation. It is relatively standard in its approach, but has the merit of sticking to experimental data. It uses state of the art knowledge of AMPAR receptor trafficking, although exclusively focusing on non-signaling pathways, and this is probably the biggest weakness of the study.`

   We agree that the signaling pathways underlying long-term plasticity and AMPAR trafficking are of general interest. However, we think that there is a misunderstanding. In fact, we consider the effects of multiple signalling pathways in our model. However, instead of modeling the pathways explicitly, we decided to integrate "only" the impacts of pathways on AMPAR trafficking by implementing the temporal development of parameter values resulting from signalling pathways during LTP (e.g., the influence of CaMKII on the binding rate; Fig. 1D and Equations. 6-8). This allowed us to keep the model concise, which simplifies mathematical analyses (e.g., Fig 5A). Please note that the explicit description of multiple signaling pathways can be integrated into our model and provides an interesting next step, which is however beyond the scope of the current manuscript.

   > (IN PAPER, pages 28:)
   >
   > **Signaling pathway models** Complex signaling pathway circuits underlie many cell processes such as synaptic plasticity. In this study, we did not model such pathways explicitly, but instead consider only their effect on AMPAR trafficking, as the time course of parameter values is determined by signalling dynamics. By this, our model becomes concise and mathematically feasible, but it does not capture complex signalling dynamics such as oscillations or switching between bistable states [18, 19]. Please note that, if affecting AMPAR trafficking, such complex signalling dynamics should be contained in the

experimentally observed time course of AMPAR exocytosis, mobility or structural plasticity being considered in this study. However, it should also be noted that the available experimental data delivers an incomplete picture such that the integration of signaling pathways models [18, 19] into our model of AMPAR trafficking could be an interesting next step to further deepen our understanding on how spine structure, enzymes and ion channels interact during LTP.

2. `Referee: Indeed, the wealth of data reporting altered AMPAR trapping during LTP for example is ignored.`

   We are aware that there is a multitude of data and related hypotheses regarding AMPAR trapping during LTP.

   In our model, we consider a temporary increase in receptor trapping following LTP induction that is possibly mediated by CaMKII and Stargazin [4, 17] (see Results, Fig4B and Table 2).

   Furthermore, in our cooperative model we also account for molecular crowding effects [20].

   Depending on the AMPAR subunit composition and/or posttranslational modifications [21], different classes of AMPA receptors could have different binding probabilities to binding sites. Thus, a class of AMPARs with higher binding probability may need to be exocytosed in the early stage of LTP to sustain potentiation. Such processes as well as the exocytosis of other molecules influencing the binding or phosphorylation state of AMPARs can be summarized as exocytic factor. While we only briefly mentioned such alternative hypotheses in the old version of the manuscript, we now included an implementation and results for an extension of our model by such an exocytic factor (see supplements) and added a section in the discussion section on this topic. Also, there exist indications that new slots may become available already during the early phase of LTP, e.g. via unbinding of SynGAP, which we mention in the discussion section. Unfortunately, we are not entirely sure, to what data exactly the referee is referring and what properties of AMPAR trapping we are ignoring but hope that those listed above are those that the referee also had in mind. Otherwise we would be thankful for some additional information.

   (IN PAPER, page 26:) However, it has been hypothesized that new binding sites may become available due to the unbinding of the protein SynGAP from PSD95 upon phosphorylation [6, 22, 23, 24]. Thereby, the generation of new slots may also contribute to E-LTP maintenance.

   (IN PAPER, page 28:)

   Exocytic factor hypothesis The dependence of LTP on exocytosis may rely on specific exocytic factors that the model does not account for. Such factors could be specific transmembrane proteins that stabilize receptors at the PSD or that increase the binding probability or even contribute to the generation of additional binding sites. Such a contribution of an exocytic factor has been hypothesized in recent reviews [25, 6]. An intriguing hypothesis is that the affinity of PSD slots for AMPARs depends on their subunit composition and/or posttranslational modifications [21]. Exocytosis of such AMPARs with different binding properties may be required for E-LTP. Such a model could

also explain E-LTP maintenance and its rapid decay in the absence of exocytosis. We show this in an exemplary model in the supplementary material (S2 Appendix).

(IN SUPPLEMENT:) As mentioned in the main text, there are other possible explanations for the dependence of E-LTP on exocytosis. One hypothesis states that due to AMPAR type specific binding affinities, exocytosis of a different AMPAR type is necessary for E-LTP expression [21]. In the following, we introduce another version of our model extended by such mechanisms and show that the resulting dynamics also match experimental results. We introduce two AMPAR types each having its own trafficking properties. These different properties may originate from a different subunit composition or phosphorylation state [21, 25].

$$\frac{dU^I}{dt} = k_{exo}\, S^I_{exo} + k_{in} + k^I_{BU}\, B - \left(k_{endo} + k_{out} + k^I_{UB}(P - (B^I + B^{II}))\right) \frac{U}{A_{spine}}. \tag{1}$$

$$\frac{dB^I}{dt} = k^I_{UB}(P - (B^I + B^{II}))\, \frac{U}{A_{spine}} - k^I_{BU} B^I. \tag{2}$$

$$\frac{dU^{II}}{dt} = k_{exo}\, S^{II}_{exo} + k^{II}_{in} + k^{II}_{BU}\, B^{II} - \left(k_{endo} + k_{out} + k^{II}_{UB}(P - (B + B^{II}))\right) \frac{U^{II}}{A_{spine}}. \tag{3}$$

$$\frac{dB^{II}}{dt} = k^{II}_{UB}(P - (B + B^{II}))\, \frac{U^{II}}{A_{spine}} - k^{II}_{BU} B^{II}. \tag{4}$$

We assume four things:

(a) Exocytosis event rates and endocytosis rates of the two receptor types are the same;

(b) receptor type II enters the spine only via exocytosis, not diffusion, i.e. we set $k^{II}_{in} = 0$;

(c) receptor type II has an increased binding affinity, i.e. $k^{II}_{UB} > k^I_{UB}$ and $k^{II}_{BU} < k^I_{BU}$;

(d) the second receptor pool is only available after LTP induction by a transition of the intracellular pool from receptor type I to type II.

The latter assumption is modeled by introducing the second receptor type to the intracellular pool (S_{exo}) in the following way:

$$\frac{dS^I_{exo}}{dt} = k^{RE}_{in} - k^{RE}_{out}\, \frac{S^I_{exo}}{V_{spine}(t)} - k_{I-II}\, S^I_{exo} + k_{II-I}\, S^{II}_{exo}, \tag{5}$$

$$\frac{dS^{II}_{exo}}{dt} = -k^{RE}_{out}\, \frac{S^{II}_{exo}}{V_{spine}(t)} + k_{I-II}\, S^I_{exo} - k_{II-I}\, S^{II}_{exo}, \tag{6}$$

where k_{I-II} and k_{II-I} are the transition rates from type I to type II and vice versa. Such a transition could reflect a long-lasting change e.g. in the receptors phosphorylation state due to increased CaMKII activity or a change in AMPAR subunit composition [26, 27]. However, the role of AMPAR phosphorylation in regulating receptor binding at the PSD is still unclear [6]. Parameter values are shown in tables Table S2A and Table S2B in S2 Text. All other parameters and the binding/unbinding rates for type I are the same as in the main text. Also, the binding rate increase for receptor type II is taken to be the same as for type I. Cooperative receptor binding and an sLTP mediated increase in exocytosis event size S_{exo} are not accounted for but spine volume increase is.

In this model, E-LTP is maintained under normal conditions whereas rapidly decaying in the absence of exocytosis (see Fig S2).

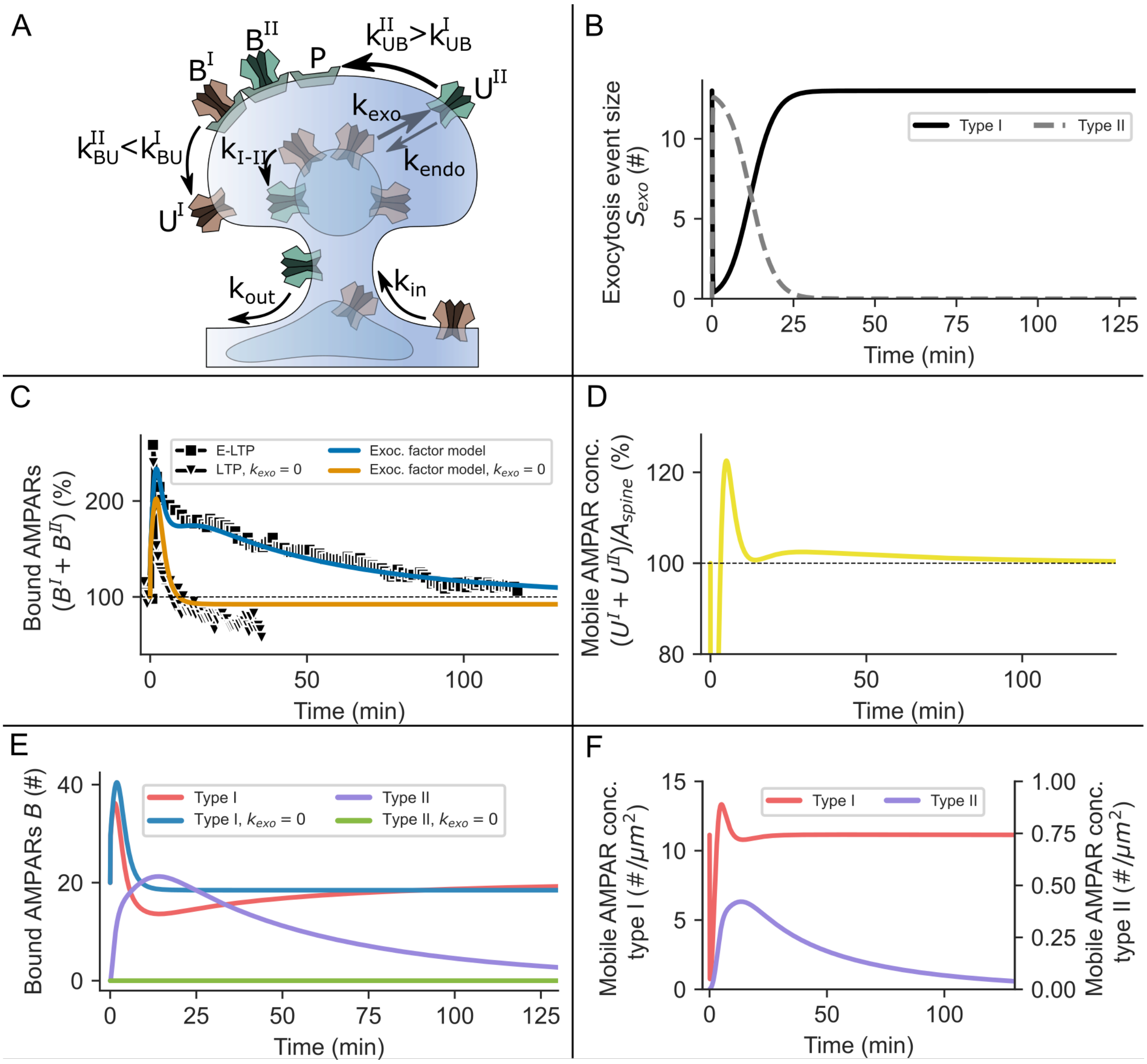

S2 Fig. E-LTP is maintained by exocytosis of a second AMPAR type having a higher binding affinity. **(A)** A second AMPAR type (green) with different binding and unbinding rates k_{UB}^{II}, k_{BU}^{II} is introduced. The availability of the second type is regulated intracellular. Receptor available for exocytosis in the intracellular pool (S_{exo}) can transition between these two types at rates k_{I-II}, k_{II-I}. **(B)** Before LTP induction, the intracellular pool for receptors to become exocytosed consists only of type I AMPARs. At the time point of LTP induction (t=0), k_{I-II} is transiently increased such that type I AMPARs (black line) are exchanged by type II AMPARs (grey line). As the transition rate k_{I-II} decays, the intracellular type II pool slowly depletes, whereas the type I pool is gradually replenished. **(C)** The transition to the exocytosis of type II AMPARs, having an increased binding affinity, mediates the expression of E-LTP (blue line). In the absence of exocytosis, type II AMPARs are absent at the spine membrane, resulting in a rapid decay of LTP (experimental data taken from [17, 16]). **(D)** The concentration of mobile receptors ($U = U^{II} + U^{I}$ rapidly increases due to the rise in the exocytosis event rate. **(E)** The initial increase in bound receptors is mediated by the rapid trapping of type I AMPARs already present at the spine surface (red and blue (blocked exocytosis) lines). Subsequent exocytosis of type II AMPARs results in a small drop of bound type I AMPARs and the accumulation of type II at the PSD

(purple line). In the absence of exocytosis, no type II AMPARs are bound (green line).**(F)** The concentration of mobile type I AMPARs initially drops due to the rapid increase in spine volume and binding rate but quickly recovers (red line). As type II AMPARs are exocytosed, their concentration increases but decays as the intracellular transition from type I to type II goes to zero **(B)**.

S2A Table. Parameters of AMPAR trafficking with two receptor types.

Parameter	Meaning	Value
k_{in}^{II}	Rate at which AMPARs enter the spine via lateral diffusion	0.0 $\#/s$
k_{BU}^{II}	Unbinding rate of receptors from PSD slots	0.025 s^{-1}
$k_{UB}^{II,0}$	Binding rate of AMPARs at the PSD	0.036 $\mu m^2/(\#s)$
k_{I-II}	Transition rate from type I to type II	0.0 s^{-1}
k_{II-I}	Transition rate from type II to type I	0.0 s^{-1}

S2B Table. LTP-induced parameter changes (two type model).

Parameter	Meaning	Value
τ_{I-II}^1	Time constant of I-II transition rate increase during LTP induction	5 s
τ_{I-II}^2	Decay time constant of I-II transition rate after LTP induction	180 s
A_{I-II}	Amount by which the transition rate increases during LTP-induction	0.25 s^{-1}

3. `Referee: While the authors are correct in stating that early signaling events such as calcium rises or CaMKII activation is only transient, AMPAR modifications could very well be long lasting and this should be taken into account and introduced as a parameter.`

   We agree that AMPAR modifications could very well be long lasting and that these modifications could be critical for receptor trapping and E-LTP maintenance. But, to our knowledge, it has yet to be shown experimentally that this is the case. We argue, that if such long lasting modifications contribute to E-LTP maintenance this contribution should depend on exocytosis. Otherwise, the fast decay of LTP in the absence of exocytosis would be difficult to explain. We include this possibility in the extended model, added to the supplement (see major point 2 above). In this extended model, a second AMPAR type with a different binding affinity is introduced. This new type could be interpreted e.g. as AMPARs with long lasting modifications in response to the transiently increased CaMKII activity. To be consistent with the fast decay of LTP in the absence of exocytosis, we assume that this new AMPAR type is only exocytosed following LTP induction.

(IN SUPPLEMENT:) Please see response to major point 2.

4. `Referee: Another point that is not explored, and should be, relates to the location of AMPAR exocytosis that is hotly debated and could be either in the spine or in the dendrite. It would be interesting to know the impact of this parameter on their model.`

The importance of the location of receptor exocytosis has been the subject of other theoretical studies (see e.g. [28, 29]). Our model does not explicitly account for spatial properties and is therefore not well suited to investigate such dependencies. However, we would argue that changing the location of exocytosis in the model results in a change in the "effective" influx of mobile receptors at the spine. If exocytosis occurs close to or at the dendrite, fewer exocytosed receptors would stay in or reach the spine respectively compared to exocytosis occurring nearby the spine's PSD. Thus, in our model different locations of exocytosis can be accounted for by changing the exocytosis event size. The dependence between exocytosis event size and bound receptors is shown in Fig 5A. While our model does not enable such investigation directly, we agree that this aspect is important and of general interest. We have therefore extended our discussion on this topic and added a paragraph, elaborating on the above thoughts, in the discussion section.

(IN PAPER, page 27:)

Location of exocytosis LTP not only influences exocytosis at the spine but also at the dendrite [30, 31]. It is however unclear whether and how receptors that are exocytosed outside the spine contribute to E-LTP expression. Theoretical studies have shown that the locus of exocytosis close to the PSD is important for synaptic capturing of AMPARs [28, 29]. A sustained increase in the exocytosis rate of GluA2 and GluA3 after LTP induction has been observed experimentally at the dendritic shaft [31]. Such a sustained local increase in dendritic receptor concentration close to a spine might increase the receptor flux (k_{in}) sufficiently to supplant or support RE mediated receptor recycling at spines and thereby contribute to E-LTP expression. As our model does not explicitly account for spatial properties, it is not well suited to investigate such dependencies. However, changing the location of exocytosis should alter the effective contribution of exocytosis to the spine's mobile pool, meaning that the fraction of the exocytic input that reaches the target site changes. If receptor exocytosis occurs close to the dendritic shaft, we expect a smaller proportion of these receptors to dwell in the spine and reach the PSD compared to exocytosis occurring at the spine. Also, as receptor binding is diffusion limited, exocytosis occurring close to the PSD would therefore increase the probability of those receptors to get bound. This effective change in the contribution of exocytosis to the receptor content at the spine could simply be accounted for by changing, for example, the exocytosis event size S_{exo} or by introducing an additional parameter that accounts for the limited availability of the input to the target site (by changing $S_{exo}\,k_{exo}$ into $S_{exo}\,k_{exo}\,b_{exo}$, where b_{exo} describes the availability of the exocytic input to the mobile receptor pool). This dependence between exocytosis event size and bound receptor number is shown in Fig 5A. Due to it's sigmoidal nature, a small shift in S_{exo} would already significantly lower the number of bound receptors in our model. Therefore, in agreement with other

theoretical studies [28, 29], our model supports the importance of exocytosis occurring close to the spine.

5. `Referee: I did not fully get the interest of their structural LTP modelling as it seems to boil down to a change in AMPAR exocytosis rate and exocytosis content. This should be further clarified.`

   It is true that sLTP mainly influences exocytosis in our model but also the receptor dwell time on the spine membrane, which should have an influence e.g. on FRAP simulations (although we did not quantify this effect). In addition, spine volume changes influence mobile receptor concentration on short time scales. More important, however, there exists little data on the dynamics of recycling endosomes and the size of exocytosis events compared to the data available on spine volume changes. However, experimental data indicates that both (membrane recycling and spine volume changes) are tightly coupled. Therefore we first formulated a model of the spine volume, which we could base on established experimental data showing the dependence on protein synthesis, exocytosis and the initial spine size. Then, we described the dependence of exocytosis on the spine volume. This model, thereby, also allows for investigation of e.g. the dependence of LTP on the initial spine size, as shown in Fig 5. We agree that these aspects are not stated clear enough in the original manuscript. We have therefore expanded the first paragraph in the results section on the sLTP model and complemented Fig 3C with examples of sLTP for different initial spine sizes. Also, we revised figure 7H and the methods section on the sLTP model.

   > (IN PAPER, pages 11:) The previous implementation of the model does not account for structural spine changes and the accompanied mobilization of recycling endosomes [11, 8, 7]. We hypothesize that, whereas the rate of exocytosis events at the spine only increases transiently (Fig 1B, [30]), the rise in recycling endosome availability during the course of sLTP results in enhanced receptor recycling and thereby in the growth of the receptor exocytosis event size S_{exo}. These assumptions are supported by experimental data showing that spine growth, membrane trafficking from recycling endosomes and AMPAR insertion are tightly coupled, all increasing about two-fold during LTP [8, 7]. We developed a reduced model in which spine volume changes (V_{spine}) are followed by a proportional but delayed change in the exocytosis event size S_{exo} (Equations 12, 13, Fig 3C). Variations in spine volume and surface area also influence the average dwell time of receptors on the spine membrane, the concentration of mobile receptors on short time scales (below equilibration time) and the total receptor surface concentration ($(U + B)/A_{spine}$). Therefore, the model composes a change in exocytosis and spine area (Equations 1,2). In addition, the dependence of sLTP on the initial spine size is accounted for [11] (Fig 3C), whose influence on E-LTP expression will be investigated later on (Fig 5, for model details see Materials and Methods). [...]

   > (IN PAPER, page 33:) In the following we describe our model of sLTP and its influence on AMPAR exocytosis from recycling endosomes at the spine [8, 7]. While much data on spine size dynamics is available, there exists little data on the recycling endosome dynamics in comparison. However, experimental data indicates that both are tightly coupled [7]. Therefore, we first formulate a model of the spine volume during sLTP and then the dependence of exocytosis (S_{exo}) on the spine volume. [...]

(IN PAPER, page 34:) [...] Furthermore, several experiments suggest that the relative increase in spine volume triggered by LTP-induction ΔV_{spine}^{long} depends on the initial spine volume V_{spine}^{0} [11, 13, 14]). The experimental data indicates that sLTP expression is strongest for small to intermediate sized spines but decays as V_{spine}^{0} increases such that there is no long-term change in volume for large spines [11]. We approximate this relationship by an exponential function:

$$\Delta V_{spine}^{long} = \frac{a \cdot e^{V_{spine}^{0}/b} + c}{100\%}, \tag{11}$$

where $a + c$ determines the maximum relative change in spine volume ΔV_{spine}^{long} and c accounts for the possibility of large spines experiencing structural depression or potentiation. b describes the decay of ΔV_{spine}^{long} with the initial spine size V_{spine}^{0}. We estimated these parameter values for three different studies [11, 13, 14] by fitting equation (11) to the experimental data. For the final model, we averaged parameters across the different estimates (values are given in the legend of Fig 7H).

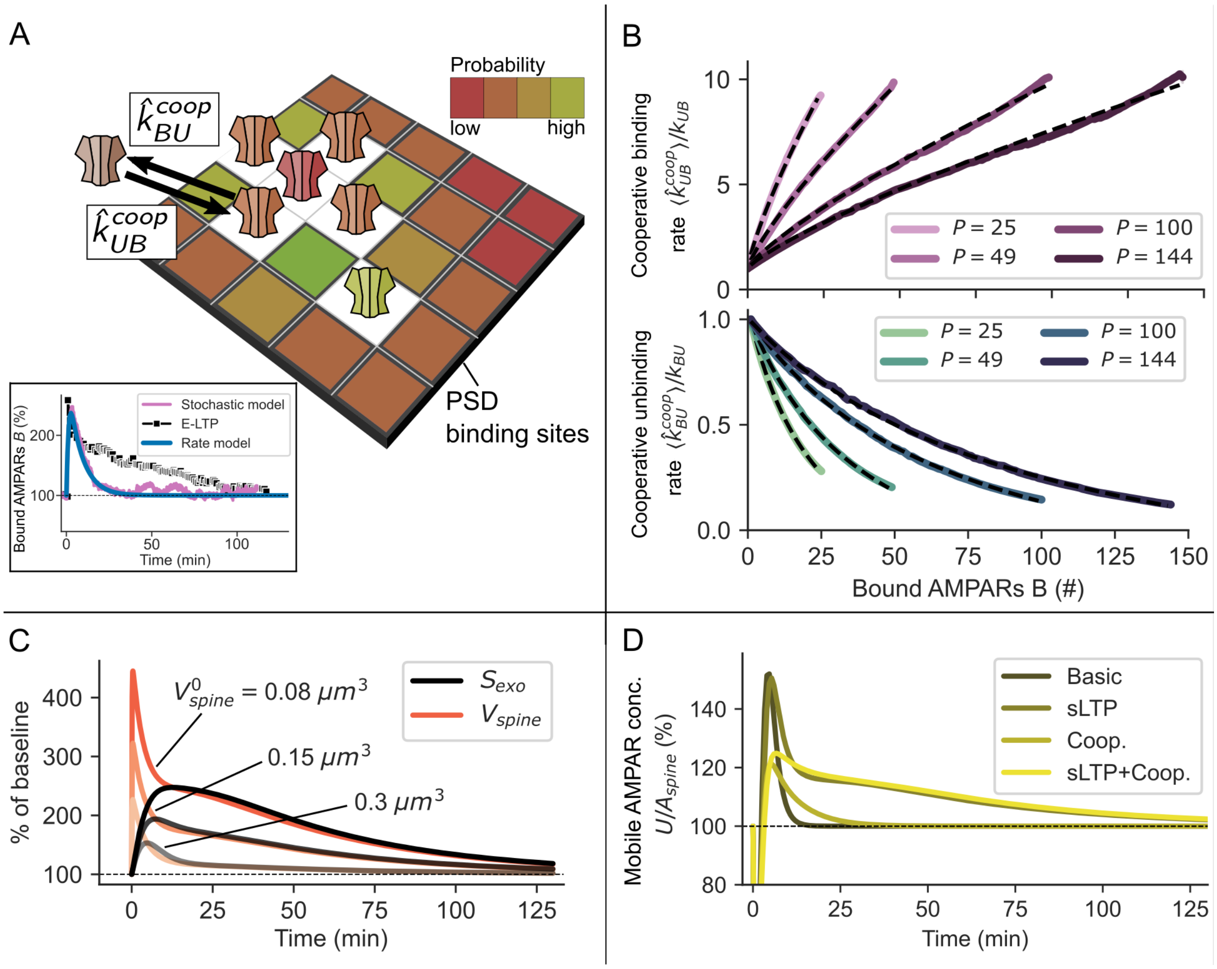

Figure 3. [...] **(C)** The enlargement of the spine during sLTP is accompanied by an increase in the size of exocytosis events S_{exo} (Equations (13) and (12)) and depends on the initial size of the spine V_{spine}^{0}. [...]

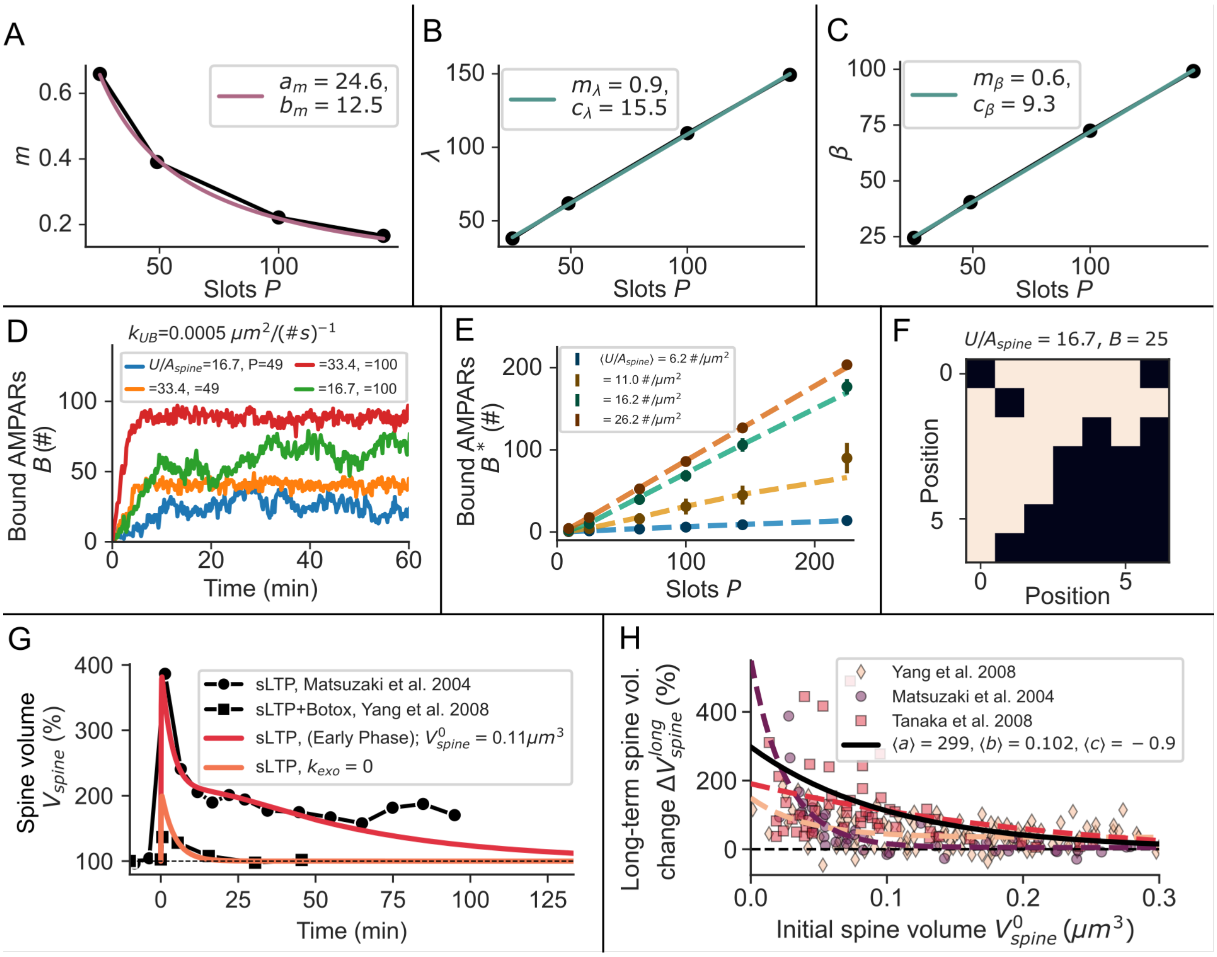

Figure 7. [...] **(H)** The relative increase in spine volume depends on the initial spine size. Dashed lines: Equation (11) has been fitted to data from [14, 11, 13] (marker). Black line: Parameters a, b, c (see Equation (11)) averaged across the individual estimates (dashed lines).

Minor points

1. `Referee: Figure 5: reference error to a non existing panel E.`

   Thank you, this should indeed be a reference to panel C not E. We have corrected the error.

2. `Referee: This statement 1346 is unclear "We, therefore, predict that cutting of the PSD from the mobile pool would result in a rapid decay of excitatory synaptic transmission."`

   Thank you for pointing this out. We have rewritten the statement in the revised manuscript.

   (IN PAPER, page 22:)

   (6) We assumed an ongoing, rapid exchange of bound receptors, which has been observed experimentally [32]. However, some receptors may be much more stable [33]. We predict that such a stable receptor pool is not necessary for E-LTP expression. This prediction could be tested experimentally by blocking exocytosis as well as diffusion into the spine to interrupt the

mobile receptor influx. If receptors are not stabilized otherwise, such depletion of the mobile receptor pool should result in the total decay of synaptic transmission within minutes.

7. `Referee: I do not understand how the authors can make the following statement l366: "Furthermore, our results indicate that sLTP accompanied by increased AMPAR tracking from REs during E-LTP"`

   We assumed an increased trafficking from REs based on the finding that AMPAR-containing REs are mobilized into the spine in parallel to spine growth and increased recycling during sLTP [8, 7]. However, we see that the motivation and origin for this assumption and the sLTP model might not have been clear enough in the original manuscript. We hope that the changes we made in response to major point 5 are sufficient to clarify this point.

   (IN PAPER) Please see major point 5.

References

[1] Shomar A, Geyrhofer L, Ziv NE, Brenner N. Cooperative stochastic binding and unbinding explain synaptic size dynamics and statistics. PLOS Computational Biology. 2017;13(7):e1005668. doi:10.1371/journal.pcbi.1005668.

[2] Nair D, Hosy E, Petersen JD, Constals A, Giannone G, Choquet D, et al. Super-Resolution Imaging Reveals That AMPA Receptors Inside Synapses Are Dynamically Organized in Nanodomains Regulated by PSD95. Journal of Neuroscience. 2013;33(32):13204–13224. doi:10.1523/jneurosci.2381-12.2013.

[3] Anggono V, Huganir RL. Regulation of AMPA receptor trafficking and synaptic plasticity. Current Opinion in Neurobiology. 2012;22(3):461–469. doi:10.1016/j.conb.2011.12.006.

[4] Lisman J, Yasuda R, Raghavachari S. Mechanisms of CaMKII action in long-term potentiation. Nature Reviews Neuroscience. 2012;13(3):169–182. doi:10.1038/nrn3192.

[5] Opazo P, Sainlos M, Choquet D. Regulation of AMPA receptor surface diffusion by PSD-95 slots. Current Opinion in Neurobiology. 2012;22(3):453–460. doi:10.1016/j.conb.2011.10.010.

[6] Choquet D. Linking Nanoscale Dynamics of AMPA Receptor Organization to Plasticity of Excitatory Synapses and Learning. The Journal of Neuroscience. 2018;38(44):9318–9329. doi:10.1523/jneurosci.2119-18.2018.

[7] Park M, Salgado JM, Ostroff L, Helton TD, Robinson CG, Harris KM, et al. Plasticity-Induced Growth of Dendritic Spines by Exocytic Trafficking from Recycling Endosomes. Neuron. 2006;52(5):817–830. doi:10.1016/j.neuron.2006.09.040.

[8] Park M, Penick EC, Edwards JG, Kauer JA, Ehlers MD. Recycling Endosomes Supply AMPA Receptors for LTP. Science. 2004;305(5692):1972–1975. doi:10.1126/science.1102026.

[9] Wang Z, Edwards JG, Riley N, Provance DW, Karcher R, dong Li X, et al. Myosin Vb Mobilizes Recycling Endosomes and AMPA Receptors for Postsynaptic Plasticity. Cell. 2008;135(3):535–548. doi:10.1016/j.cell.2008.09.057.

[10] Zheng N, Jeyifous O, Munro C, Montgomery JM, Green WN. Synaptic activity regulates AMPA receptor trafficking through different recycling pathways. eLife. 2015;4. doi:10.7554/elife.06878.

[11] Matsuzaki M, Honkura N, Ellis-Davies GCR, Kasai H. Structural basis of long-term potentiation in single dendritic spines. Nature. 2004;429(6993):761–766. doi:10.1038/nature02617.

[12] Matsuzaki M, Ellis-Davies GCR, Nemoto T, Miyashita Y, Iino M, Kasai H. Dendritic spine geometry is critical for AMPA receptor expression in hippocampal CA1 pyramidal neurons. Nature Neuroscience. 2001;4(11):1086–1092. doi:10.1038/nn736.

[13] Tanaka J, Horiike Y, Matsuzaki M, Miyazaki T, Ellis-Davies GCR, Kasai H. Protein Synthesis and Neurotrophin-Dependent Structural Plasticity of Single Dendritic Spines. Science. 2008;319(5870):1683–1687. doi:10.1126/science.1152864.

[14] Yang Y, b Wang X, Frerking M, Zhou Q. Spine Expansion and Stabilization Associated with Long-Term Potentiation. Journal of Neuroscience. 2008;28(22):5740–5751. doi:10.1523/jneurosci.3998-07.2008.

[15] Bosch M, Castro J, Saneyoshi T, Matsuno H, Sur M, Hayashi Y. Structural and Molecular Remodeling of Dendritic Spine Substructures during Long-Term Potentiation. Neuron. 2014;82(2):444–459. doi:10.1016/j.neuron.2014.03.021.

[16] Barco A, Alarcon JM, Kandel ER. Expression of Constitutively Active CREB Protein Facilitates the Late Phase of Long-Term Potentiation by Enhancing Synaptic Capture. Cell. 2002;108(5):689–703. doi:10.1016/s0092-8674(02)00657-8.

[17] Penn AC, Zhang CL, Georges F, Royer L, Breillat C, Hosy E, et al. Hippocampal LTP and contextual learning require surface diffusion of AMPA receptors. Nature. 2017;549(7672):384–388. doi:10.1038/nature23658.

[18] Kotaleski JH, Blackwell KT. Modelling the molecular mechanisms of synaptic plasticity using systems biology approaches. Nature Reviews Neuroscience. 2010;11(4):239–251. doi:10.1038/nrn2807.

[19] Smolen P, Baxter DA, Byrne JH. Molecular Constraints on Synaptic Tagging and Maintenance of Long-Term Potentiation: A Predictive Model. PLoS Computational Biology. 2012;8(8):e1002620. doi:10.1371/journal.pcbi.1002620.

[20] Kokolaki ML, Fauquier A, Renner M. Molecular Crowding and Diffusion-Capture in Synapses. iScience. 2020;23(8):101382. doi:10.1016/j.isci.2020.101382.

[21] Diering GH, Huganir RL. The AMPA Receptor Code of Synaptic Plasticity. Neuron. 2018;100(2):314–329. doi:10.1016/j.neuron.2018.10.018.

[22] Nicoll RA. A Brief History of Long-Term Potentiation. Neuron. 2017;93(2):281–290. doi:10.1016/j.neuron.2016.12.015.

[23] Araki Y, Zeng M, Zhang M, Huganir RL. Rapid Dispersion of SynGAP from Synaptic Spines Triggers AMPA Receptor Insertion and Spine Enlargement during LTP. Neuron. 2015;85(1):173–189. doi:10.1016/j.neuron.2014.12.023.

[24] Walkup WG, Mastro TL, Schenker LT, Vielmetter J, Hu R, Iancu A, et al. A model for regulation by SynGAP-α1 of binding of synaptic proteins to PDZ-domain Slots in the postsynaptic density. eLife. 2016;5. doi:10.7554/elife.16813.

[25] Hiester BG, Becker MI, Bowen AB, Schwartz SL, Kennedy MJ. Mechanisms and Role of Dendritic Membrane Trafficking for Long-Term Potentiation. Frontiers in Cellular Neuroscience. 2018;12. doi:10.3389/fncel.2018.00391.

[26] Huganir RL, Nicoll RA. AMPARs and Synaptic Plasticity: The Last 25 Years. Neuron. 2013;80(3):704–717. doi:10.1016/j.neuron.2013.10.025.

[27] Lee SJR, Escobedo-Lozoya Y, Szatmari EM, Yasuda R. Activation of CaMKII in single dendritic spines during long-term potentiation. Nature. 2009;458(7236):299–304. doi:10.1038/nature07842.

[28] Kusters R, Kapitein LC, Hoogenraad CC, Storm C. Shape-Induced Asymmetric Diffusion in Dendritic Spines Allows Efficient Synaptic AMPA Receptor Trapping. Biophysical Journal. 2013;105(12):2743–2750. doi:10.1016/j.bpj.2013.11.016.

[29] Czondor K, Mondin M, Garcia M, Heine M, Frischknecht R, Choquet D, et al. Unified quantitative model of AMPA receptor trafficking at synapses. Proceedings of the National Academy of Sciences. 2012;109(9):3522–3527. doi:10.1073/pnas.1109818109.

[30] Patterson MA, Szatmari EM, Yasuda R. AMPA receptors are exocytosed in stimulated spines and adjacent dendrites in a Ras-ERK-dependent manner during long-term potentiation. Proceedings of the National Academy of Sciences. 2010;107(36):15951–15956. doi:10.1073/pnas.0913875107.

[31] Tanaka H, Hirano T. Visualization of Subunit-Specific Delivery of Glutamate Receptors to Postsynaptic Membrane during Hippocampal Long-Term Potentiation. Cell Reports. 2012;1(4):291–298. doi:10.1016/j.celrep.2012.02.004.

[32] Heine M, Groc L, Frischknecht R, que JCB, Lounis B, Rumbaugh G, et al. Surface Mobility of Postsynaptic AMPARs Tunes Synaptic Transmission. Science. 2008;320(5873):201–205. doi:10.1126/science.1152089.

[33] Makino H, Malinow R. AMPA Receptor Incorporation into Synapses during LTP: The Role of Lateral Movement and Exocytosis. Neuron. 2009;64(3):381–390. doi:10.1016/j.neuron.2009.08.035.
